# 11例原发性皮肤弥漫大B细胞淋巴瘤，腿型的临床特征和疗效分析

**DOI:** 10.3760/cma.j.issn.0253-2727.2023.08.015

**Published:** 2023-08

**Authors:** 跃兴 袁, 晴 施, 洋 赫, 惠玲 裘, 红梅 易, 磊 董, 黎 王, 澍 程, 彭鹏 许, 维莅 赵

**Affiliations:** 1 上海交通大学医学院附属瑞金医院血液科，医学基因组学国家重点实验室，上海血液学研究所，上海 200025 State Key Laboratory of Medical Genomics, Shanghai Institute of Hematology, Shanghai Rui Jin Hospital, Shanghai Jiao Tong University School of Medicine, Shanghai 200025, China; 2 福建医科大学附属龙岩第一医院血液风湿科，龙岩 364000 Department of Hematology and Rheumatology, Longyan First Hospital Affiliated to Fujian Medicine University, Longyan 364000, China

原发性皮肤弥漫大B细胞淋巴瘤，腿型（PCDLBCL，LT）多发于老年患者，是一种罕见的侵袭性非霍奇金淋巴瘤（NHL），在每年新发原发性皮肤淋巴瘤中约占4％[1]。研究表明，PCDLBCL，LT患者中存在MYD88、PIM1、CD79B等基因的高频突变，且BCR信号通路上的基因（CD79A/B或CARD11）突变与患者的不良预后相关[2]。目前，国内对于PCDLBCL，LT的研究仅见于个别病例报告及小样本量的临床病理研究，尚未开展对PCDLBCL，LT的分子病理学研究。为了进一步明确中国PCDLBCL，LT患者的临床病理特征和疗效，本文收集了11例PCDLBCL，LT患者的临床资料并进行了相关研究。

## 病例与方法

1. 病例：收集上海交通大学医学院附属瑞金医院自2014年8月至2022年3月收治的11例诊断为PCDLBCL，LT的患者。完善血常规、生化常规、心电图、骨髓穿刺、病理、全身CT或PET-CT等检查，所有患者均经皮肤病理及免疫组织化学染色确诊，诊断参照WHO-EORTC分类标准[3]。

2. 治疗方案与疗效评估：大部分患者接受了以R-CHOP方案（利妥昔单抗+环磷酰胺+阿霉素+长春新碱+泼尼松）为基础的治疗方案，疗效评估参照2014年Lugano会议修订标准[4]，包括完全缓解（CR）、部分缓解（PR）、疾病稳定（SD）、疾病进展（PD）。总有效率（ORR）为CR率与PR率之和。

3. 靶向测序：利用石蜡包埋组织基因组DNA提取试剂盒（德国QIAGEN公司产品）提取患者肿瘤组织石蜡切片中的gDNA，取200 ng gDNA，利用预文库构建试剂盒［艾吉泰康生物科技（北京）有限公司］构建预文库，利用靶向测序试剂盒对目的基因（55个淋巴瘤相关基因，分别为ARID1A、ATM、B2M、BCL6、BTG1、BTG2、CARD11、CCND3、CD58、CD70、CD79A、CD79B、CIITA、CREBBP、DDX3X、DTX1、DUSP2、EBF1、EP300、EZH2、FAS、FBXW7、FOXO1、GNA13、HIST1H1C、HIST1H1E、IRF4、IRF8、KMT2C、KMT2D、LYN、MAPK7、MPEG1、MTOR、MYC、MYD88、NFKBIE、NOTCH1、NOTCH2、PIM1、PRDM1、PTPN6、SGK1、SOCS1、STAT3、STAT6、TBL1XR1、TET2、TMSB4X、TNFAIP3、TNFRSF14、TP53、TSC2、ZFP36L1、ZNF608）进行液相杂交捕获并构建文库，采用Novaseq测序平台（美国Illumina公司产品）进行测序。测序由上海睿昂基因科技股份有限公司辅助完成，测序完成后，将原始测序序列与人类参考基因组hg19进行比对，将重复序列剔除，重新校准碱基质量后，利用GATK进行SNP calling并使用ANNOVAR进行分析。过滤掉以下突变：①在genomAD、千人基因组数据库中频率高于0.001的突变；②位于非编码区的突变；③同义突变；④被SIFT、Polyphen2、CADD等生物信息学软件预测为无害的错义突变。

4. 随访：整理患者的住院病历资料，于2022年6月20日对所有患者进行电话随访，确认患者的疾病进展状态及生存状态，中位随访时间为38.5（2.4～68.0）个月。随访截止日期为2022年6月。总生存（OS）期指患者自确诊到死亡或随访截止的时间，无进展生存（PFS）期指患者自确诊到疾病进展、复发、死亡或随访截止的时间。

5. 统计学处理：应用SPSS软件进行数据分析，计数资料以例数（百分比）表示，计量资料用中位数（范围）或*x*±*s*表示。Fisher精确检验用于评估两组间CR率的差异，Kaplan-Meier法、Log-rank检验和单因素Cox比例风险模型进行生存分析。

## 结果

1. 临床特征：本研究共纳入11例PCDLBCL，LT患者，其中男8例（62.7％），女3例（27.3％），男女比例为2.7∶1；患者中位年龄为72岁，其中9例（81.8％）患者年龄大于60岁；有B症状患者2例（18.2％）；生发中心来源（GCB）患者1例（9.1％），非生发中心来源（non-GCB）患者10例（90.9％）；Ann Arbor分期Ⅰ～Ⅱ期患者6例（54.5％），Ⅲ～Ⅳ期患者5例（45.5％）；7例（63.6％）患者乳酸脱氢酶（LDH）含量升高；7例（63.6％）患者美国东部肿瘤协作组体力状况（ECOG）评分为0～1分，4例（36.4％）患者ECOG评分≥2分；所有患者均结外受累，其中6例（54.5％）患者下肢受累，7例（63.6％）患者结外受累个数为1，2例（18.2％）患者结外受累个数为2，2例（18.2％）患者结外受累个数大于2；国际预后指数（IPI）评分0～2分的患者5例（45.5％），6例（54.5％）患者IPI评分≥3分。

2. 疗效：11例PCDLBCL，LT患者中7例（63.6％）接受以R-CHOP方案为基础的治疗方案，均获得CR；3例（27.3％）接受泽布替尼联合来那度胺及利妥昔单抗（ZR2）方案，其中2例患者获得CR，1例患者获得PR；1例（9.1％）未接受化疗，予口服沙利度胺、维A酸治疗。应用R-CHOP方案患者的CR率为100％，应用ZR2治疗方案患者的CR率为66.7％，两者的差异无统计学意义（*P*＝0.300）。

3. 生存分析：11例PCDLBCL，LT患者的中位随访时间为38.5（2.4～68.0）个月，随访期间4例（36.4％）患者PD，3年PFS率为（61.4±15.3）％。7例接受R-CHOP方案治疗患者的3年PFS率为（57.1±18.7）％，3例接受ZR2方案治疗患者的3年PFS率为（66.7±27.2）％。随访期间2例（18.2％）患者死亡，3年OS率为（80.8±12.2）％。7例接受R-CHOP方案治疗患者的3年OS率为（85.7±13.2）％，3例接受ZR2方案治疗患者的3年OS率为（50.0±35.4）％。

4. 突变分析：靶向测序结果显示，9例PCDLBCL，LT患者中共检出21个基因突变，突变频率≥20％的基因包括PIM1（67％）、MYD88（44％）、TMSB4X（33％）、TBL1XR1（33％）、KMT2D（33％）、CD79B（33％）、CARD11（33％）、BTG2（33％）、BTG1（22％）（图1）。

**图1:**
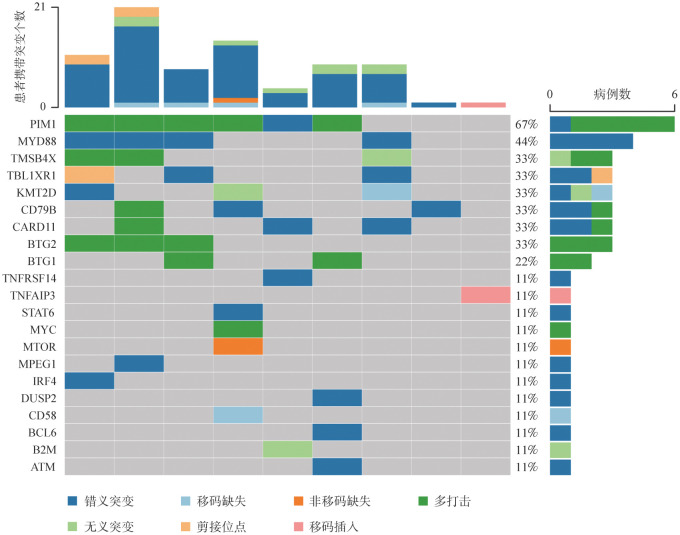
9例原发性皮肤弥漫大B细胞淋巴瘤，腿型患者的基因突变图谱

5. 预后的单因素分析：单因素分析结果显示，KMT2D基因突变与患者较短的OS期有相关趋势，但差异无统计学意义（*P*＝0.083）（表1）。B症状与患者较短的PFS期有相关趋势，但差异无统计学意义（*P*＝0.065）（表1）。

**表1:** 影响11例原发性皮肤弥漫大B细胞淋巴瘤，腿型患者预后的单因素分析

因素	总生存期			无进展生存期	
	*HR*（95% *CI*）	*P*值		*HR*（95% *CI*）	*P*值
性别（女，男）	0.028（0～Inf）	0.581		0.024（0～141.448）	0.401
B症状（有，无）	6.000（0.365～98.720）	0.218		6.423（0.891～46.302）	0.065
Ann Arbor分期（Ⅲ～Ⅳ，Ⅰ～Ⅱ）	81.456（0.001～Inf）	0.452		5.052（0.517～49.341）	0.164
LDH（升高，正常）	40.046（0～Inf）	0.551		1.936（0.201～18.652）	0.568
ECOG评分（≥2分，<2分）	1.871（0.117～29.956）	0.658		1.735（0.242～12.439）	0.583
结外病变数（≥2，<2）	1.479（0.092～23.879）	0.783		1.818（0.254～13.020）	0.552
IPI评分（≥3分，<3分）	54.178（0.001～Inf）	0.495		3.102（0.321～30.005）	0.328
Hans分型（non-GCB，GCB）	23.670（0～Inf）	0.759		24.362（0～Inf）	0.643
KMT2D突变（有，无）	434.450（0～Inf）	0.083		1.891（0.160～22.290）	0.613

注 LDH：乳酸脱氢酶；ECOG：美国东部肿瘤协作组体力状况；IPI：国际预后指数；non-GCB：非生发中心来源；GCB：生发中心来源；Inf：无穷大

## 讨论

PCDLBCL，LT十分罕见，临床中需要与其他累及皮肤的淋巴瘤进行鉴别，尤其是原发性皮肤滤泡中心淋巴瘤（PCFCL）。国外的一项研究表明，PCFCL与GCB型弥漫大B细胞淋巴瘤（DLBCL）基因表达谱相似，而PCDLBCL，LT与ABC型DLBCL基因表达谱相似[5]，进行CD10、BCL6、MUM-1等免疫表型的检测有助于二者的鉴别。在本研究中non-GCB型患者比例高达90.9％，仅有1例GCB型患者，表明了PCDLBCL，LT与non-GCB型DLBCL的相关性。GCB型PCDLBCL，LT在国外也有少量报道[6]–[7]。国外数据显示，PCDLBCL，LT多发于老年患者[8]，国内2篇报道均显示男性患者数量高于女性患者[9]–[10]。本研究中11例PCDLBCL，LT患者的中位年龄72岁，女性患者占27.3％，和相关研究报道一致[9]–[10]。R-CHOP方案显著改善了PCDLBCL，LT患者的预后，使患者的3年生存率从不足50％提升至80％[11]。本研究中7例接受R-CHOP方案治疗患者的3年OS率为（85.7±13.2）％，与文献报道相近，而3例接受ZR2方案治疗患者的3年OS率为（50.0±35.4）％。尽管受限于样本量，该结果表明以R-CHOP方案为基础的治疗可能使中国PCDLBCL，LT患者受益。

研究报道，MYD88（69％～75％）、PIM1（69％～70％）、CD79B（40％～56％）、HIST1H1E（41％）、TBL1XR1（33％）、CREBBP（26％）、MYC（20％～26％）、IRF4（16％）、KMT2D（15％）和CARD11（5％）是PCDLBCL，LT患者的常见突变基因，这些基因在B细胞信号转导和NF-κB活化中发挥重要作用[2],[12]。本研究同样检测了PIM1、MYD88、TBL1XR1、CD79B、CARD11、MYC等基因的突变情况，突变频率与既往报道接近。此外，研究发现基因的突变状态与PCDLBCL，LT患者的预后显著相关。如BCR信号通路基因（CD79A/B或CARD11）的突变与PCDLBCL，LT患者PFS期和OS期缩短相关，且携带该基因突变的患者会出现治疗抵抗[2]。由于样本量的限制，本研究探究了突变频率≥20％基因的突变状态与患者预后的关系，结果提示KMT2D突变与患者较短的OS期有相关趋势。KMT2D基因编码的蛋白在表观遗传调控中发挥重要作用，累积的证据显示，KMT2D突变与多种淋巴瘤的预后相关。Ferrero等[13]发现携带KMT2D突变的套细胞淋巴瘤患者预后较差，病情恶化和死亡风险升高。Li等[14]发现，在血浆循环肿瘤DNA中检测到KMT2D突变的NK/T细胞淋巴瘤患者预后较差。Khanam等[15]发现KMT2D突变的淋巴母细胞淋巴瘤患者的复发率显著升高。但本研究未发现CD79B和CARD11基因的突变状态与PCDLBCL，LT患者的OS和PFS具有相关性，可能与PCDLBCL，LT的异质性及样本量较少有关。

此外，本研究结果提示B症状与PCDLBCL，LT患者PFS期缩短相关，但差异无统计学意义。既往与PCDLBCL，LT相关的研究均未关注患者是否出现B症状，但针对中国淋巴瘤患者的研究显示B症状与多种淋巴瘤的预后相关。周智俊等[16]发现B症状是影响原发性乳腺淋巴瘤患者预后的因素；杨渤彦等[17]发现B症状与DLBCL患者的OS率显著相关等。上述结果进一步表明B症状与PCDLBCL，LT患者的预后可能存在相关性。

综上所述，本研究探究了11例PCDLBCL，LT患者的临床特征，初步展示了中国PCDLBCL，LT患者的突变图谱。本研究结果显示KMT2D突变和B症状可能是影响中国PCDLBCL，LT患者预后的因素，可能为PCDLBCL，LT患者的治疗和预后评估提供新的思路。
